# Relative biological effectiveness of therapeutic proton beams for HSG cells at Japanese proton therapy facilities

**DOI:** 10.1093/jrr/rru003

**Published:** 2014-04-03

**Authors:** Mizuho Aoki-Nakano, Yoshiya Furusawa, Akiko Uzawa, Yoshitaka Matsumoto, Ryoichi Hirayama, Chizuru Tsuruoka, Takashi Ogino, Teiji Nishio, Kazufumi Kagawa, Masao Murakami, Go Kagiya, Kyo Kume, Masanori Hatashita, Shigekazu Fukuda, Kazutaka Yamamoto, Hiroshi Fuji, Shigeyuki Murayama, Masaharu Hata, Takeji Sakae, Hideki Matsumoto

**Affiliations:** 1 Research Center for Particle Therapy, National Institute of Radiological Sciences, 4-9-1 Anagawa, Inage-ku, Chiba 263-8555, Japan; 2 National Cancer Center Hospital East, 6-5-1 Kashiwanoha, Kashiwa, Chiba 277-8577, Japan; 3 Hyogo Ion Beam Medical Center, 1-2-1 Koto, Shingu-cho, Tatsuno-shi, Hyogo 679-5165, Japan; 4 Wakasa Wan Energy Research Center, 64-52-1 Nagatani, Tsuruga, Fukui 914-0192, Japan; 5 Shizuoka Cancer Center, 1007 Shimonagakubo, Nagaizumi-cho, Sunto-gun, Shizuoka 411-8777, Japan; 6 Proton Medical Research Center, University of Tsukuba, 2-1-1 Amakubo, Tsukuba, Ibaraki 365-8576, Japan; 7 University of Fukui, 23-3 Matsuoka-shimoaizuki, Eiheiji-cho, Yoshida-gun, Fukui 910-1193, Japan; 8 Proton Therapy Center, Fukui Prefectural Hospital, 2-8-1, Yotsui, Fukui 910-8526, Japan

**Keywords:** proton therapy, relative biological effectiveness, cell survival, spread-out Bragg-peak

## Abstract

We investigated the relative biological effectiveness (RBE) of therapeutic proton beams at six proton facilities in Japan with respect to cell lethality of HSG cells. The RBE of treatments could be determined from experimental data. For this purpose, we used a cell survival assay to compare the cell-killing efficiency of proton beams. Among the five linear accelerator (LINAC) X-ray machines at 4 or 6 MeV that were used as reference beams, there was only a small variation (coefficient of variation CV = 3.1% at D_10_) in biological effectiveness. The averaged value of D_10_ for the proton beams at the middle position of the spread-out Bragg peak (SOBP) was 4.98. These values showed good agreement, with a CV of 4.3% among the facilities. Thus, the average RBE_10_ (RBE at the D_10_ level) at the middle position of the SOBP beam for six facilities in Japan was 1.05 with a CV of 2.8%.

## INTRODUCTION

Particle radiotherapy is currently an attractive option for oncologists as a new and promising modality for treating malignant tumors. The Heavy-Ion Medical Accelerator in Chiba (HIMAC) facility was constructed in 1993, and clinical trials with carbon beams were initiated there in June 1994, together with academic research activities, including those related to treatment, diagnosis, physics, engineering and biology. Following the HIMAC cancer therapy facility, several ion beam therapy facilities were set up in Japan in the 2000s. We had performed radiobiological studies involving ion beams prior to the initiation of the HIMAC clinical trials [1–3]. We had also performed a relative biological effectiveness (RBE) study at new ion beam facilities, such as the Hyogo Ion Beam Medical Center, the Wakasa Wan Energy Research Center and the Shizuoka Cancer Center, before patient treatment started [4, 5].

New proton treatment facilities are emerging, and previous data on RBE is very useful to them because they can obtain the RBE values obtained at existing Japanese facilities using the same biological systems. However, the standard range for RBE has not been published. Here, we present a unified method for radiobiological testing, coupled with the results of previous RBE studies. This report may not provide new scientific insights, but it may provide important and useful information for new facilities. We have described a variation of RBE and other parameters at a number of therapeutic proton facilities to confirm whether RBE shows good agreement between the facilities.

## MATERIALS AND MTEHODS

### Cell culture

A human salivary gland tumour (HSG) cell line (JCRB1070: HSGc-C5, Japanese Collection of Research Bioresources Cell Bank) was used in this study. The cell line is a standard reference cell line of RBE used at the NIRS (National Institute of Radiological Sciences)-HIMAC in carbon beam research. The cells were subcultured according to the method described below and were stored in liquid nitrogen at a concentration of 1 × 10^6^ cells/ml in the complete culture medium described below with 10% DMSO. The cells were subcultured in a bottle twice a week until a final concentration of 1 × 10^4^ cells/cm^2^ was obtained and were then used for experiments within 20 passages after purchase from JCRB to obtain stable results.

Cells were cultured in Eagle's minimum essential medium (E-MEM; SIGMA, M4655) supplemented with 10% fetal bovine serum (FBS) and antibiotics (100 U/ml penicillin and 100 µg/ml streptomycin) under humidified conditions with 5% CO_2_ and 95% air at 37°C. The FBS was tested before use such that the delay in growth after seeding was <12 h. Exponentially growing cells were trypsinized and seeded in plastic flasks (NUNC 152094 or other flasks without a slanted neck) at 2 × 10^4^ cells/cm^2^ and incubated for 1 d prior to the irradiation at each facility.

### Irradiation

All proton 6-cm spread-out Bragg peak (SOBP) beams were generated from 180–235 MeV beams. High-energy LINAC X-rays at 4 or 6 MeV were used as the reference at each facility, and the energy depended on the specific instrumentation of each facility. The samples were placed at the isocenter of the SOBP beam, and the depth was adjusted to be in the middle position of the SOBP beam. The cells were then irradiated at room temperature. Dosimetry was performed according to the standard protocol by IAEA [6] for both proton and X-ray beams. Briefly, either Type 30001 Farmer Chamber or Type 23343 Markus Chamber (PTW, Freiburg) was placed at sample position. Monitor chambers placed upstream of the sample were calibrated by output from the above-stated chambers. Cell samples were irradiated using the preset values of the monitor chamber. A summary of the proton and X-ray beams for each facility is given in Table 1.

**Table 1.** Summary of facilities and D_10_, D_37_, SF_2_ and RBEs

Facility	A	B	C	D	E	F	Average	CV (%)
Year of experiment	2000	2001	2002	2002	2002	2011		
Proton energy (MeV)	190	180	190	190	200	235		
Dose rate (Gy/min)	∼1.5	∼2.0	1.6–2.7	1.0–1.5	0.9–1.3	∼3.0		
Reference X-ray (MeV)	4	4	6	4		4		
Proton D_10_ (Gy)	5.1	4.97	4.91	5.32	4.92	4.68	4.98	4.3
Proton D_37_ (Gy)	2.87	2.44	2.72	3.09	2.9	2.44	2.74	9.5
Proton SF_2_	0.655	0.557	0.687	0.707	0.628	0.458	0.615	15.1
X-ray D_10_ (Gy)	5.25	5.29	5	5.42	^b^	5.1	5.21	3.1
X-ray D_37_ (Gy)	2.88	2.97	2.88	3.13	^b^	2.87	2.95	3.8
X-ray SF_2_	0.655	0.655	0.621	0.704		0.541	0.635	9.5
RBE_10_^a^	1.03 ± 0.06	1.06 ± 0.05	1.02 ± 0.06	1.02 ± 0.05	1.07 ± 0.06	1.09 ± 0.03	1.05	2.8
RBE_37_^a^	1.00 ± 0.05	1.22 ± 0.14	1.06 ± 0.05	1.01 ± 0.06	1.02 ± 0.07	1.18 ± 0.10	1.08	8.6

The width of the SOBP was 60 mm for all facilities. ^a^RBE at 10% and 37% survival levels; values are the average and SD for each facility. ^b^There was no High-energy LINAC X-ray machine available at facility E. Averaged values from facilities A–D were used in the RBE calculation.

### Colony formation assay

Irradiated cells were kept in the dark at a low temperature until they were moved to the laboratory. The cells were rinsed twice with PBS(-) and trypsinised (0.2% trypsin in PBS(-)) to harvest them and were then resuspended in the culture medium. After the cell concentration was determined with a particle analyzer (Coulter counter), cells were diluted adequately and plated onto triplicate 6-cm plastic dishes, aiming for 100 colonies per dish for the cell survival assay. After 13 days' incubation in the CO_2_ incubator, the colonies were rinsed with PBS(-) once, fixed with 10% formalin solution for 10–15 min, and stained with 1% methylene blue. Any colony consisting of >50 cells was counted under a stereomicroscope as a surviving colony.

Survival curves were fitted using the linear–quadratic (LQ) model equation: SF = exp (-αD-βD^2^), where SF is the surviving fraction and D is the physical dose at the sample position. The parameters α and β were calculated with the least squares method described below. The α and β values were used to recalculate the biological equivalent dose D_10_ and D_37_ values, that is, the doses at which the cell survival is 10% and 37%, respectively, and the SF_2_ values after 2-Gy exposure. RBE_10_ values were obtained from a comparison of the D_10_ value for both proton and X-ray beams. The RBE values of proton beams were calculated from the X-ray data in each facility. The α and β values were obtained by the least square method. At least three independent experiments were performed using both proton beams and X-rays at each facility.

## RESULTS AND DISCUSSION

**Fig. 1.**
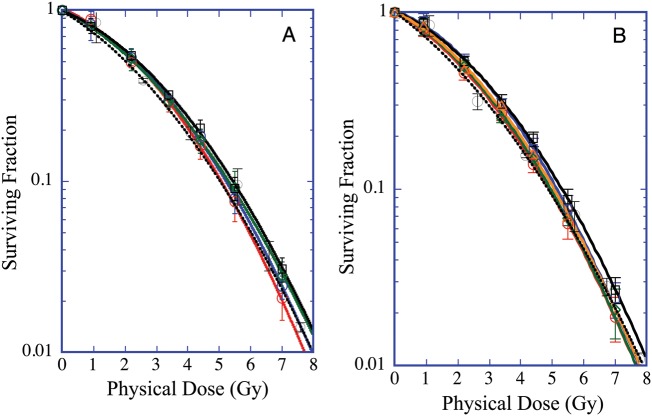
Survival curves of HSG cells obtained **A**) from LINAC high-energy X-ray machines at five different therapy facilities located at the corresponding proton facility, and **B**) at the middle position of 6-cm SOBP beams at six different proton therapy facilities. The red, blue, green, black, brown and broken lines correspond to each facility A to F, respectively. The symbols and bars are the mean and the standard deviation, respectively, obtained from at least three independent experiments.

The survival curves relative to the experiments conducted with X-rays and the proton SOBP beam for each facility are shown in Figs 1A and B. The survival curves from each facility are almost identical. In the case of X-rays, the D_10_ and D_37_ values on HSG cells varied among facilities between 5.00 and 5.42 Gy and between 2.87 and 3.13Gy, respectively (Table 1), with averaged values of 5.21 Gy (3.1% coefficient of variation, CV) and 2.95 Gy (3.8% CV), respectively. Similarly, in the case of proton SOBP beams, average values of D_10_ and D_37_ were 4.98 Gy (4.3% CV) and 2.74 (9.5% CV) Gy, respectively.

The RBE_10_ values that were calculated from the survival curves (Table 1) showed good agreement among the facilities, with a 2.8% CV. The average RBE_10_ at the middle of the proton SOBP obtained from all the facilities was 1.05 ± 0.03. Similar to the value obtained from another study, the RBE value at the middle of the 6-cm SOBP in the crypt survival assay ranged from 1.01–1.05 *in vitro* or 1.01–1.08 *in vivo* [5] or from 1.0–1.11 *in vivo* [7].

The experimental RBE_10_ (RBE values at D_10_) values of the proton SOBP beams from each facility, obtained from the cell survival assay performed in this study, ranged from 1.02–1.09, while a representative clinical RBE value of either 1.0 or 1.1 is used in proton treatment facilities worldwide [8]. Experimental RBE differs from clinical RBE, and the RBE of a given type of radiation will vary with particle type and energy, dose, dose per fraction, degree of oxygenation, cell or tissue type, biological endpoint, etc. [9]. There must be differences between expected clinical RBE and experimental RBE, and the clinical RBE of a carbon beam at HIMAC was determined based on both biological experiments with neutron and carbon beams, and clinical results from neutron therapy conducted at NIRS [10]. The experimental RBEs varied from generic clinical RBE [11, 12]. Thus the RBE_10_ that we have obtained in this study (1.05) was smaller than the representative clinical RBE value of 1.1. Generic RBE [12] is an averaged value from many radiobiological experiments including different cells and different endpoints. RBE in this paper is a specific value of cell killing at D_10_ level on HGS cells.

The highest values of D_10_, D_37_ and SF_2_ were found in facility D for both proton and X-ray irradiation. However, these values were in good agreement with those of the other facilities with acceptable variance (except for SF_2_). Surprisingly, the range of the survival parameter values (averaged values ± SD or CV) at facility F was similar to that of the other facilities and in the range of CVs, even though the experiment was conducted 10 years after those at the other facilities, the cells were older, serum and other chemicals were purchased from different manufacturers, and some of our experimental staff had changed.

## CONCLUSION

D_10_ values of X-rays among the five therapy facilities in Japan showed good agreement, with deviations of 5.21 Gy with 3.1% CV and ranged from 5.0–5.42 Gy. Similarly, proton beams at six facilities also showed D_10_ values of 4.98 Gy with 4.3% CV and ranged from 4.68–5.32 Gy. The RBE_10_ values of the six facilities were identical, and the averaged value was 1.05 ± 0.03 with a small deviation (2.8% CV).
